# Association between the prognostic nutritional index and impulse control disorders in patients with early-stage Parkinson's disease

**DOI:** 10.3389/fnut.2026.1863248

**Published:** 2026-07-17

**Authors:** Wen Zhou, Jiao-jiao Shi, Duan Liu, Tian-fang Zeng, Qing-qing Xia

**Affiliations:** 1 West China School of Medicine, Sichuan University Affiliated Chengdu Second People's Hospital, Chengdu Second People's Hospital, Sichuan University, Chengdu, Sichuan, China; 2 Department of Nutrition, General Hospital of the Western Theater Command, Chengdu, Sichuan, China

**Keywords:** impulse control disorders, neuroinflammation, Parkinson's disease, PPMI, prognostic nutritional index

## Abstract

**Background:**

Impulse control disorders (ICDs) are common non-motor symptoms in Parkinson's disease (PD) and associated with significant psychiatric morbidity and reduced quality of life. The prognostic nutritional index (PNI) reflects both nutritional and immune status. However, the relationship between PNI and ICDs in early-stage PD remains unknown.

**Methods:**

This cross-sectional study included 1,302 early-stage PD patients (Hoehn and Yahr stages 1–3) from the Parkinson's Progression Markers Initiative (PPMI) database. Logistic regression models were constructed with sequential adjustment for demographic, clinical, neuropsychiatric, metabolic, and laboratory covariates. Sensitivity analyses included complete-case analysis, exclusion of participants with abnormal liver or renal function, and propensity score-based methods (IPTW, SMRW, PA). Receiver operating characteristic (ROC) curves, net reclassification improvement (NRI), and integrated discrimination improvement (IDI) were calculated to assess incremental predictive value.

**Results:**

The overall prevalence of ICDs was 22.6% (294/1,302). In the fully adjusted model, each 1-unit increase in PNI was associated with 5% lower odds of ICDs (OR = 0.95, 95% CI: 0.92–0.98, *P* = 0.003). Compared with the lowest PNI tertile (T1), the highest tertile (T3) showed 34% reduced odds of ICDs (OR = 0.66, 95% CI: 0.46–0.94, *P* = 0.023), with a significant dose-response trend (*P* for trend = 0.025). Subgroup analyses revealed no significant interactions in any strata. Adding PNI to the baseline model significantly improved category-free reclassification (continuous NRI = 0.14, *P* = 0.03404; IDI = 0.0069, *P* = 0.00497), although the increase in discrimination was modest and non-significant (C-statistic from 0.714 to 0.720, *P* = 0.223). All sensitivity analyses confirmed the robustness of these association.

**Conclusions:**

Higher PNI is independently associated with lower odds of ICDs in early-stage PD patients. PNI, a simple and readily available composite marker of nutritional and immune status, may serve as a valuable adjunctive tool for discriminating PD patients with higher odds of prevalent ICDs. Prospective longitudinal studies are warranted to establish causality and explore potential nutritional or immunomodulatory interventions.

## Introduction

Parkinson's disease (PD) is a common neurodegenerative disorder primarily characterized by the progressive loss of dopaminergic neurons in the substantia nigra pars compacta. This degeneration underlies the classic motor symptoms of PD, including resting tremor, rigidity, bradykinesia, and postural instability. However, accumulating clinical and fundamental research has increasingly recognized that non-motor symptoms (NMSs) are equally prevalent in PD and often highly disabling, significantly impairing patients' quality of life (1). Among these NMSs, impulse control disorders (ICDs) represent a particularly concerning category of psychiatric and behavioral disturbances, encompassing pathological gambling, hypersexuality, compulsive eating, and compulsive buying (2). In addition, some patients may exhibit related behaviors such as punding, hobbyism, and uncontrolled walking or driving.

Epidemiological evidence indicates that the overall prevalence of ICDs among PD patients is approximately 30%, which is substantially higher than that observed in the general population (3–5). Moreover, multiple studies have demonstrated that PD patients with ICDs experience more severe non-motor symptoms than those without addictive behaviors, particularly neuropsychiatric problems such as depression, reduced quality of life, impaired working memory, and a higher frequency of rapid eye movement sleep behavior disorder (3, 6, 7). Depression not only coexists with ICDs but has also been shown to increase the risk of developing ICDs in PD patients (8, 9).

From a pathophysiological perspective, oxidative stress and neuroinflammation are considered core drivers of dopaminergic neurodegeneration in PD. Of note, neuroimmune abnormalities have also been widely documented in various psychiatric disorders, including major depressive disorder, bipolar disorder, schizophrenia, and obsessive-compulsive disorder (10). As part of the impulse control spectrum, ICDs have similarly shown potential links to immuno-inflammatory pathways (11). Against this background, identifying simple and reliable peripheral blood biomarkers that reflect neuroinflammatory status is of great significance for the early recognition and intervention of ICDs in PD patients. In recent years, several peripheral blood cell-based inflammatory markers, such as the systemic immune-inflammation index (SII) and the systemic inflammation response index (SIRI), have been shown to be associated with NMSs like anxiety in PD (12). In addition, the uric acid-to-creatinine ratio (UA/Cr) has been found to correlate with specific ICD subtypes and the coexistence of multiple ICDs in PD (13). However, no study to date has specifically examined the relationship between the prognostic nutritional index (PNI) and ICDs in PD patients. PNI is a composite indicator calculated from serum albumin level and peripheral blood lymphocyte count (serum albumin + 0.005 × lymphocyte count). It reflects both nutritional status and integrated immune-inflammatory information and has demonstrated predictive value in various neuropsychiatric conditions.

Therefore, the present study aims to investigate the association between PNI and ICDs in PD patients, with the goal of providing new clinical evidence for the neuroinflammatory mechanisms underlying PD-related impulse control disorders and exploring the potential utility of PNI as a feasible biomarker.

## Methods

### Study population and data source

All data used in the present study were obtained from the Parkinson's Progression Markers Initiative (PPMI) database, RRID: SCR_006431. The data were downloaded on December 18, 2025. PPMI is a large-scale, multicenter, observational study designed to identify biomarkers of PD progression. The database includes comprehensive clinical, imaging, and biospecimen data from participants across multiple international sites. All participants provided written informed consent, and the study protocol was approved by the institutional review board (IRB) at each participating site. The present study involved the analysis of de-identified, publicly available data; therefore, additional ethical approval was not required by our institution.

**Figure 1:**
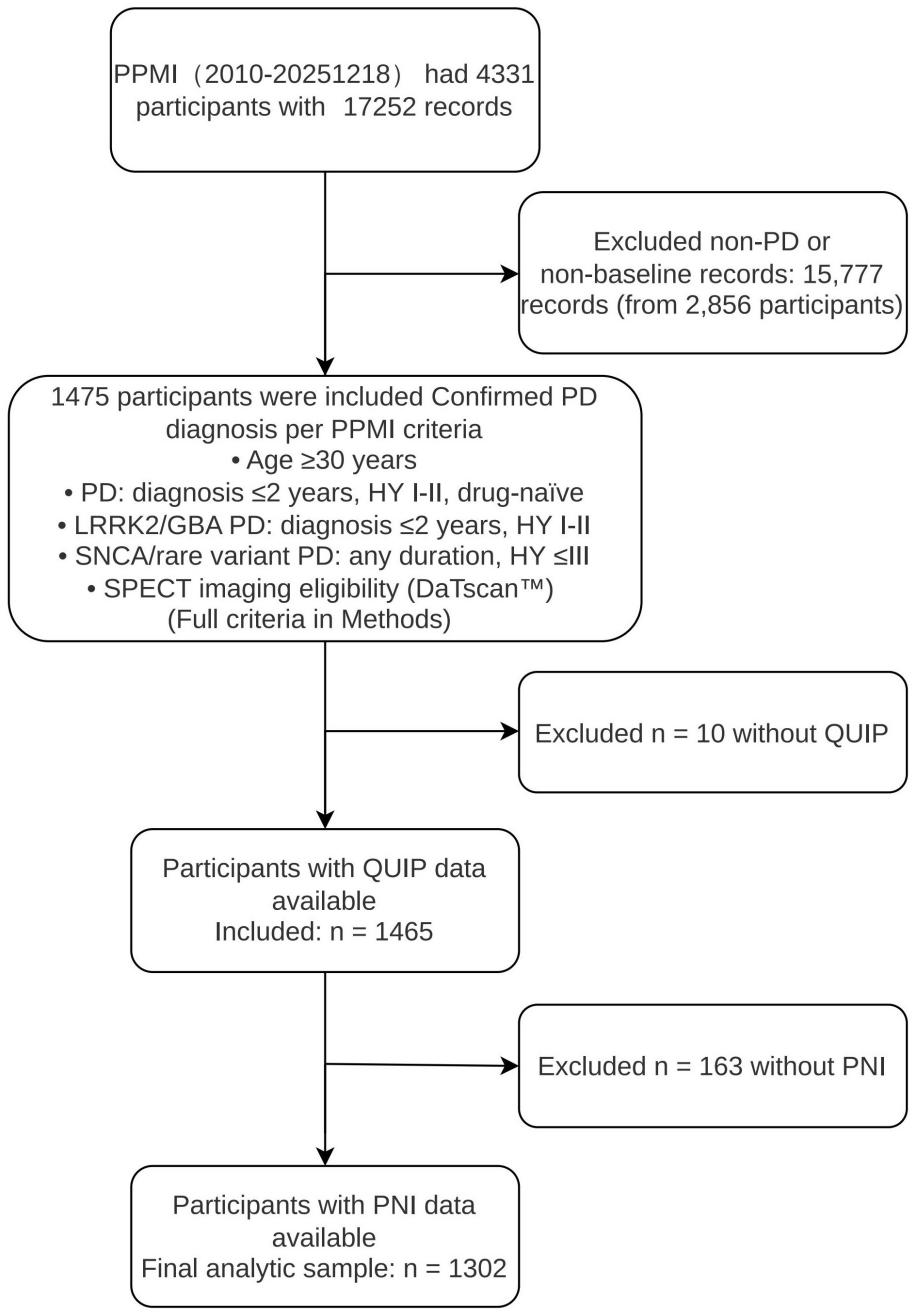
Flowchart of participant selection from the Parkinson's Progression Markers Initiative (PPMI) cohort. The PPMI database (2010–2025) initially contained 4,331 participants with 17,252 records. Non-PD or non-baseline records (15,777 records from 2,856 participants) were excluded, leaving 1,475 participants who met the PPMI diagnostic criteria for Parkinson's disease (PD). Key PD criteria varied by genetic subgroup: PD (age ≥ 30 years, diagnosis ≤ 2 years at screening, drug-naïve at baseline [no PD medication within 60 days and cumulative use ≤ 90 days], Hoehn & Yahr stage I–II, typical motor features, and SPECT eligibility); LRRK2/GBA PD (similar clinical criteria including diagnosis ≤ 2 years and HY I–II, but without the drug-naïve requirement); SNCA or rare genetic variant PD (any disease duration, HY up to III, no restriction on prior or ongoing PD medication). All participants underwent DaT SPECT imaging for diagnostic confirmation, and standard PPMI exclusion criteria applied to all groups. Full diagnostic criteria are described in the Methods section. After applying QUIP availability, 10 participants without QUIP data were excluded, resulting in 1,465 participants with complete QUIP data. Subsequently, 163 participants without complete PNI data (albumin and lymphocyte count) were excluded, yielding a final analytic sample of 1,302 participants. QUIP, Questionnaire for Impulsive-Compulsive Disorders in Parkinson's Disease; PNI, Prognostic Nutritional Index.

This was a cross-sectional analysis using baseline data only. We included baseline data from participants with a confirmed diagnosis of PD. The detailed participant selection process is illustrated in Figure 1. The diagnosis of PD was established according to the standardized PPMI clinical protocol (14). Eligible PD participants met the following inclusion criteria: (i) the initial early PD cohort required diagnosis within 2 years of screening, Hoehn & Yahr (HY) stage I–II at baseline, no expected need for PD medication for at least 6 months, no use of levodopa/dopamine agonists/MAO-B inhibitors/amantadine within 60 days prior to baseline and cumulative use ≤ 90 days, plus typical motor features (resting tremor, bradykinesia, rigidity, or asymmetry) and confirmation by DaT SPECT; (ii) patients with LRRK2 or GBA variants were enrolled using similar clinical criteria (HY I–II, disease duration ≤ 2 years, motor features) but without the medication-naïve or off-medication requirement; (iii) patients with SNCA or other rare genetic variants (e.g., Parkin, PINK1) were included regardless of disease duration, allowed HY stage up to III, and had no restriction on prior or ongoing PD medication use. All participants underwent DaT SPECT imaging for diagnostic confirmation, and standard PPMI exclusion criteria (e.g., atypical parkinsonism, dementia, use of dopamine receptor blockers within 6 months, contraindications to lumbar puncture) applied to all groups.

The following data were obtained from the PPMI database for each participant: (1) demographic and lifestyle factors: age, sex, race, education years, and body mass index (BMI); (2) disease-related characteristics: age at PD onset, genetic subtype (sporadic vs. genetic), Unified Parkinson's Disease Rating Scale Part III score (UPDRS III), HY stage; (3) neuropsychiatric and cognitive measures: Montreal Cognitive Assessment (MoCA) score, REM sleep behavior disorder (RBD) score, Geriatric Depression Scale (GDS) score; (4) metabolic and laboratory parameters: serum uric acid, aspartate aminotransferase (AST), alanine aminotransferase (ALT), creatinine, albumin, lymphocyte count; (5) comorbidities: hyperlipidemia (HLP), hypertension (HP), diabetes mellitus (DM); (6) imaging: bilateral striatal binding ratio (SBR) derived from DaTscan; (7) outcome variable: presence of any ICDs assessed by the Questionnaire for Impulsive-Compulsive Disorders in Parkinson's Disease-Short Form (QUIP-SF). The outcome variable was the presence of impulse control disorders (ICDs) assessed by the QUIP-SF, a validated screening instrument for ICDs and related disorders in PD. The QUIP evaluates seven domains: (i) buying, (ii) eating, (iii) gambling, (iv) hobbies (dopamine dysregulation syndrome), (v) punding, (vi) sex, and (vii) walking/driving. Each domain is scored as 0 (no disorder) or 1 (disorder present). The primary outcome was defined as the presence of any 1 or more disorders (quip any = 1) vs. no disorders (quip any = 0); and (8) medication status: use of any anti-Parkinsonian medication (yes/no), defined as a levodopa equivalent daily dose (LEDD) > 0.

PNI was calculated as serum albumin (g/L) + 5 × total lymphocyte count (10^9^/L), where lymphocyte counts reported as × 10^3^/μL in the PPMI dataset were converted to 10^9^/L (1 × 10^3^/μL = 1 × 10^9^/L). Per PPMI protocol, all research blood samples were collected after a minimum 8-h fast.

### Statistical analysis

Normality was assessed using histograms, Q-Q plots, and the Kolmogorov–Smirnov test. Continuous variables are expressed as mean ± SD or median (IQR) according to their distribution, and categorical variables as frequencies (%). Group comparisons were performed using the independent-samples *t*-test or Mann-Whitney U test for continuous data, and the chi-square or Fisher's exact test for categorical data, as appropriate.

To handle missing entries, we performed multiple imputation using chained equations (R mice package, five repetitions) (15). All primary analyses were carried out on these imputed datasets. We used logistic regression to evaluate how PNI relates to ICDs. PNI entered the models first as a continuous measure, then split into tertiles to compute a trend P-value, which helped assess linearity and possible non-linear patterns. To visualize dose-response curves, we fitted cubic splines with four knots placed at the 5th, 35th, 65th and 95th percentiles. Non-linearity was formally examined by a likelihood ratio test, comparing a model with only a linear term against one that included both linear and spline terms. When a non-linear relationship was detected, we further applied a two-piecewise linear regression to pinpoint a threshold.

Covariate selection drew on three complementary sources: (i) prior literature identifying established ICD risk factors (8, 13, 16–22); (ii) clinical and biological reasoning regarding potential confounders of the PNI–ICD association; and (iii) empirical screening, specifically variables associated with the outcome at *P* < 0.05 in univariate analysis or whose inclusion altered the PNI effect estimate by >10%. We built four hierarchical adjustment models. Model 1 had no covariate adjustment (crude model). Model 2 added demographic and lifestyle factors: age, sex, education years, race, and BMI. Model 3 further incorporated on top of Model 2 the following: disease-related features (PD medication use, age at onset, UPDRS III score, and SBR) and neuropsychiatric and cognitive status (MoCA, RBD, and GDS). Model 4 (full model) additionally adjusted for metabolic and laboratory measures: HLP, HP, DM, serum uric acid, AST, and creatinine.

To evaluate the robustness of our findings, we conducted several sensitivity analyses. These included: (1) repeating all analyses on complete cases (i.e., listwise deletion of missing values); (2) excluding participants with abnormal liver function, defined as any AST or ALT value outside the normal reference range (either below or above the laboratory-defined limits); (3) excluding participants with abnormal creatinine values (outside the normal range); and (4) to further minimize potential confounding bias, we applied a series of propensity score-based methods, including propensity score matching (PSM) (23), inverse probability of treatment weighting (IPTW) (24), standardized mortality ratio weighting (SMRW) (25), and pairwise algorithmic weighting (PA) (26). For these analyses, PNI was dichotomised at the median (low PNI vs. high PNI) to define the binary exposure variable. The propensity score was estimated using logistic regression predicting low PNI (vs. high PNI) based on all covariates in Model 4 (age, sex, education years, race, BMI, PD medication use, age at onset, UPDRS III score, MoCA, GDS, RBD, SBR, HLP, HP, DM, AST, creatinine, and serum uric acid). For PSM, genetic matching with a caliper width of 0.2 was used to achieve balanced covariate distributions between compared groups. Standardized mean differences (SMDs) were calculated to assess the balance of baseline covariates in both the unmatched and matched samples. An SMD greater than 0.1 or a two-sided *P* value less than 0.05 was considered indicative of significant covariate imbalance. The results of these propensity score-based analyses were compared with the primary findings to confirm the robustness of the association between PNI and ICDs.

Prespecified subgroup analyses were performed to explore whether the association between PNI and ICDs varied across different strata, including age (<65 vs. ≥65 years), BMI (<25, 25–30, ≥30 kg/m^2^), sex, HP, DM, HLP, age at onset (<50 vs. ≥50 years), PD medication use, and genetic subtype (sporadic vs. genetic). For subgroup analyses, the interaction *P* values were corrected for multiple testing using the Benjamini-Hochberg false discovery rate (FDR) method. An FDR-adjusted *P* < 0.05 was considered statistically significant.

We evaluated whether adding PNI improved the discriminative performance of conventional models using ROC curve analysis. For each participant, we calculated the area under the ROC curve (AUC)—equivalent to the C-statistic—to quantify how well the logistic models could discriminate ICD cases from non-cases. Two sets of models were fitted: first, with PNI as a continuous variable; second, with PNI categorized into tertiles. For each specification, we constructed a baseline model containing all covariates from our full adjustment set (Model 4 without PNI) and an extended model that additionally included PNI (either continuously or as tertiles). The DeLong method was used to test whether the AUCs of the two models differed significantly. To further quantify how much PNI added beyond established factors, we computed the continuous net reclassification improvement (NRI) and the integrated discrimination improvement (IDI) for the continuous PNI model. Bootstrap resampling (500 replicates) provided 95% confidence intervals for these incremental indices. Statistical significance was set at a two-sided *P* < 0.05.

All statistical computations were carried out using R version 4.2.2 and the Free Statistics platform (version 1.9), a Python-based graphical user interface that utilizes R as the underlying computational engine. A two-sided *P* value <0.05 was considered statistically significant.

## Results

### Baseline characteristics of study participants

A total of 1,302 patients with Parkinson's disease were included after applying the selection criteria. Of these, the overall prevalence of any ICD was 22.6% (294/1,302). The mean PNI level of the entire cohort was 44.0 ± 3.5. Baseline characteristics stratified by PNI tertiles are summarized in Table 1 using observed (complete-case) data; numbers that do not add up to 100% are attributable to missing data. Significant differences across PNI tertiles were observed for age, age at onset, GDS score, RBD score, striatal binding ratio, prevalence of hyperlipidemia, albumin, lymphocytes, and PNI itself (all *P* < 0.05). No significant differences were found for sex, race, BMI, education years, genetic subtype, H-Y stage, UPDRS III score, MoCA score, prevalence of hypertension, prevalence of diabetes mellitus, AST, ALT, serum uric acid, or PD medication use (all *P* > 0.05).

**Table 1:** Baseline characteristics of study participants according to PNI tertiles.

Variables	PNI				*p*
	Total (*n* = 1,302)	T1 (*n* = 430)	T2 (*n* = 433)	T3 (*n* = 439)	
ICD, *n* (%)					0.039
No	1,008 (77.4)	325 (75.6)	325 (75.1)	358 (81.5)	
Yes	294 (22.6)	105 (24.4)	108 (24.9)	81 (18.5)	
Age, Mean ± SD	62.9 ± 9.7	64.8 ± 9.1	63.0 ± 9.7	60.9 ± 9.9	<0.001
Sex, *n* (%)					0.645
Female	499 (38.3)	166 (38.6)	172 (39.7)	161 (36.7)	
Male	803 (61.7)	264 (61.4)	261 (60.3)	278 (63.3)	
Race, *n* (%)					0.909
White	1223 (94.4)	405 (94.4)	402 (93.3)	416 (95.4)	
Black	13 (1.0)	5 (1.2)	5 (1.2)	3 (0.7)	
Asian	18 (1.4)	6 (1.4)	7 (1.6)	5 (1.1)	
Other	42 (3.2)	13 (3)	17 (3.9)	12 (2.8)	
BMI, Mean ± SD	26.9 ± 4.9	27.0 ± 4.7	26.5 ± 4.9	27.2 ± 5.0	0.093
Education years, Mean ± SD	15.8 ± 3.1	15.8 ± 3.0	15.7 ± 3.2	15.8 ± 3.1	0.871
Age onset, Mean ± SD	60.0 ± 10.2	62.1 ± 9.3	60.1 ± 10.1	57.8 ± 10.6	<0.001
Genetic subtype, *n* (%)					0.517
Sporadic PD	983 (75.5)	331 (77)	319 (73.7)	333 (75.9)	
Genetic PD	319 (24.5)	99 (23)	114 (26.3)	106 (24.1)	
H-Y, *n* (%)					0.68
1	426 (34.2)	134 (32.8)	146 (35.1)	146 (34.6)	
2	797 (63.9)	264 (64.5)	264 (63.5)	269 (63.7)	
3	24 (1.9)	11 (2.7)	6 (1.4)	7 (1.7)	
UPDRS III score, Mean ± SD	22.4 ± 10.1	22.6 ± 9.8	22.6 ± 10.4	22.1 ± 10.2	0.702
MoCA, Mean ± SD	26.8 ± 2.6	26.7 ± 2.7	26.8 ± 2.6	26.9 ± 2.6	0.613
GDS, Median (IQR)	2.0 (0.0, 3.0)	2.0 (1.0, 4.0)	2.0 (0.0, 3.0)	2.0 (0.0, 3.0)	0.089
RBD, Median (IQR)	3.0 (2.0, 6.0)	4.0 (2.0, 6.0)	4.0 (2.0, 6.0)	3.0 (2.0, 5.0)	<0.001
SBR, Mean ± SD	0.8 ± 0.2	0.8 ± 0.2	0.8 ± 0.2	0.8 ± 0.3	0.006
HLP, *n* (%)					0.007
No	928 (71.3)	284 (66)	312 (72.1)	332 (75.6)	
Yes	374 (28.7)	146 (34)	121 (27.9)	107 (24.4)	
HP, *n* (%)					0.354
No	872 (67.0)	278 (64.7)	290 (67)	304 (69.2)	
Yes	430 (33.0)	152 (35.3)	143 (33)	135 (30.8)	
DM, *n* (%)					0.818
No	1212 (93.1)	401 (93.3)	405 (93.5)	406 (92.5)	
Yes	90 (6.9)	29 (6.7)	28 (6.5)	33 (7.5)	
AST (U/L), Mean ± SD	22.1 ± 9.2	22.1 ± 12.0	22.4 ± 7.7	21.9 ± 7.3	0.729
ALT (U/L), Mean ± SD	21.7 ± 12.5	20.1 ± 12.0	21.8 ± 12.4	23.1 ± 13.0	0.002
Creatinine (μmol/L), Mean ± SD	82.1 ± 17.9	84.3 ± 20.2	81.6 ± 17.6	80.4 ± 15.6	0.005
Serum Uric Acid (μmol/L), Mean ± SD	303.8 ± 77.2	304.8 ± 74.9	297.9 ± 76.5	308.6 ± 79.8	0.118
Albumin (g/L), Mean ± SD	44.0 ± 3.5	40.6 ± 2.4	44.4 ± 2.0	46.9 ± 2.6	<0.001
Lymphocytes (× 10^3^/uL), Mean ± SD	1.6 ± 0.6	1.4 ± 0.3	1.5 ± 0.4	2.0 ± 0.7	<0.001
PNI, Mean ± SD	52.1 ± 4.5	47.4 ± 2.2	52.0 ± 1.1	56.9 ± 2.8	<0.001
LEDD (mg/d) among medicated patients, Median (IQR)	–	–	–	–	–
Only for patients with PD medication (n=264)	450.0 (296.9, 800.0)	540.6 (300.0, 933.8)	400.0 (250.0, 692.0)	450.0 (262.0, 742.4)	0.089
PD medication use, *n* (%)					0.679
No	1,038 (79.7)	348 (80.9)	340 (78.5)	350 (79.7)	
Yes	264 (20.3)	82 (19.1)	93 (21.5)	89 (20.3)	

PNI tertiles were defined based on the distribution in the study population: T1, <50.1; T2, 50.1–53.85; T3, ≥ 53.85. ICD, presence of any impulse control disorder assessed by the Questionnaire for Impulsive-Compulsive Disorders in Parkinson's Disease (QUIP); PD, Parkinson's disease; n, number; ALT, alanine aminotransferase; AST, aspartate aminotransferase; BMI, body mass index; DM, diabetes mellitus; GDS, Geriatric Depression Scale; HLP, hyperlipidemia; HP, hypertension; SBR, bilateral striatal binding ratio derived from DaTscan; MoCA, Montreal Cognitive Assessment; PNI, prognostic nutritional index; RBD, rapid eye movement sleep behavior disorder (assessed by the REM Sleep Behavior Disorder Questionnaire); SD, standard deviation; H-Y, Hoehn and Yahr Scale; UPDRS III score, Unified Parkinson's Disease Rating Scale Part III score; LEDD, Levodopa equivalent daily dose. Only calculated for patients receiving anti-parkinsonian medication (*n* = 264, 20.3% of the total cohort). For unmedicated patients (LEDD = 0), the median (IQR) would be 0 (0, 0), which is not informative and therefore omitted from the table. Numbers that do not add up to 100% are attributable to missing data.

### Association between PNI and ICDs

After full adjustment for potential confounders, each 1-unit increase in PNI was associated with 5% lower odds of prevalent ICDs (OR = 0.95, 95% CI: 0.92–0.98, *P* = 0.003). When PNI was examined by tertiles, individuals in the highest tertile (T3) showed 34% lower odds of prevalent ICDs relative to those in the lowest tertile (T1) (OR = 0.66, 95% CI: 0.46–0.94, *P* = 0.023). A clear dose-response trend of decreasing ICD prevalence across increasing PNI tertiles was evident (*P* for trend = 0.025) (Table 2, Model 4).

**Table 2:** Association between prognostic nutritional index (PNI) and impulse control disorders (ICDs) in patients with early-stage Parkinson's disease.

Variable	*N* total	*N* event %	Model 1		Model 2		Model 3		Model 4	
			OR (95%CI)	*P* value	OR (95%CI)	*P* value	OR (95%CI)	*P* value	OR (95%CI)	*P* value
PNI	1,302	294 (22.6)	0.96 (0.93~0.99)	0.009	0.95 (0.92~0.98)	0.003	0.95 (0.92~0.98)	0.003	0.95 (0.92~0.98)	0.003
T1	430	105 (24.4)	1 (Ref)		1 (Ref)		1 (Ref)		1 (Ref)	
T2	433	108 (24.9)	1.03 (0.75~1.4)	0.858	1.02 (0.74~1.4)	0.911	1.02 (0.73~1.43)	0.892	1.02 (0.73~1.42)	0.924
T3	439	81 (18.5)	0.7 (0.51~0.97)	0.032	0.65 (0.47~0.91)	0.012	0.67 (0.47~0.95)	0.024	0.66 (0.46~0.94)	0.023
Trend test	1,302	294 (22.6)		0.035		0.013		0.026		0.025

Model 1: crude model with no covariate adjustment.

Model 2: adjusted for age, sex, education years, race, and body mass index.

Model 3: additionally adjusted for PD medication use, age at onset, UPDRS III score, striatal binding ratio, MoCA score, RBD score, and GDS score.

Model 4: further adjusted for hyperlipidemia, hypertension, diabetes mellitus, serum uric acid, AST, and creatinine.

PNI tertiles were defined based on the distribution in the study population: T1, <50.1; T2, 50.1–53.85; T3, ≥ 53.85. CI, confidence interval; GDS, Geriatric Depression Scale; ICD, impulse control disorder; MoCA, Montreal Cognitive Assessment; OR, odds ratio; PD, Parkinson's disease; PNI, prognostic nutritional index; RBD, rapid eye movement sleep behavior disorder; UPDRS, Unified Parkinson's Disease Rating Scale; AST, aspartate aminotransferase.

### Dose-response relationship between PNI and ICDs

**Figure 2:**
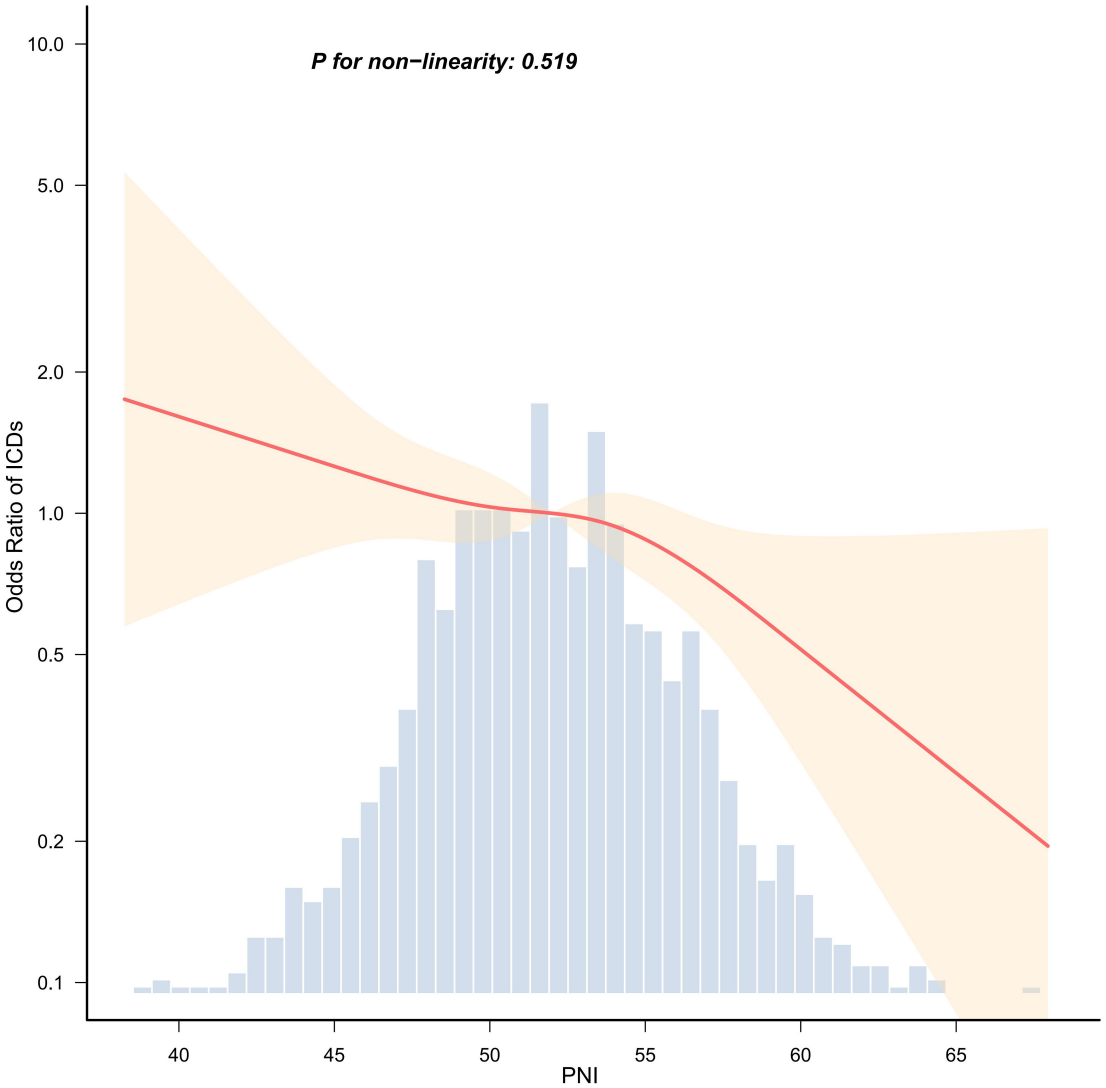
Dose-response relationship between PNI and the odds of ICDs. Cubic spline regression with four knots at the 5th, 35th, 65th and 95th percentiles of the PNI distribution. The solid line represents the estimated odds ratio, and the shaded area indicates the 95% confidence interval. The model was adjusted for all covariates in Model 4. The *P* value for non-linearity was 0.519, indicating no significant departure from linearity.

As shown in Figure 2, there was a progressive decline in the odds of ICDs with increasing PNI levels. The test for non-linearity was not statistically significant (*P* for non-linearity = 0.519), indicating that a linear model adequately captured the inverse relationship between PNI and ICDs. Accordingly, no threshold effect was further explored using two-piecewise linear regression.

### Sensitivity analyses

To assess the robustness of the association between PNI and ICDs, we performed several sensitivity analyses, including complete-case analysis, exclusion of participants with abnormal liver or renal function, and propensity score-based methods. The results of these sensitivity analyses are summarized in Table 3.

**Table 3:** Sensitivity analyses for the association between prognostic nutritional index (PNI) and impulse control disorders (ICDs) in patients with early-stage Parkinson's disease.

Variable	*N* total	*N* event %	Model 1		Model 2		Model 3		Model 4	
			OR (95%CI)	*P* value	OR (95%CI)	*P* value	OR (95%CI)	*P* value	OR (95%CI)	*P* value
Exclusion of participants with any missing data	1,170.0	258 (22.1)	0.96 (0.93~0.99)	0.015	0.95 (0.92~0.99)	0.006	0.95 (0.92~0.99)	0.005	0.95 (0.92~0.99)	0.005
Exclusion of participants with abnormal liver function	1,217.0	267 (21.9)	0.96 (0.93~0.99)	0.005	0.95 (0.92~0.98)	0.001	0.95 (0.91~0.98)	0.002	0.94 (0.91~0.98)	0.002
Exclusion of participants with abnormal creatinine values	1,277.0	291 (22.8)	0.96 (0.93~0.99)	0.006	0.95 (0.92~0.98)	0.002	0.95 (0.92~0.98)	0.002	0.95 (0.92~0.98)	0.002
IPTW									0.75 (0.57~0.97)	0.028
SMRW									0.74 (0.57~0.97)	0.027
PA									0.73 (0.55~0.97)	0.032

Model 1: crude model with no covariate adjustment.

Model 2: adjusted for age, sex, education years, race, and body mass index.

Model 3: additionally adjusted for PD medication use, age at onset, UPDRS III score, striatal binding ratio, MoCA score, RBD score, and GDS score.

Model 4 (full model): further adjusted for hyperlipidemia, hypertension, diabetes mellitus, serum uric acid, AST, and creatinine.

AST, aspartate aminotransferase; ALT, alanine aminotransferase; CI, confidence interval; GDS, Geriatric Depression Scale; ICD, impulse control disorder; IPTW, inverse probability of treatment weighting; MoCA, Montreal Cognitive Assessment; OR, odds ratio; PA, pairwise algorithmic weighting; PD, Parkinson's disease; PNI, prognostic nutritional index; RBD, rapid eye movement sleep behavior disorder; SMRW, standardized mortality ratio weighting; UPDRS, Unified Parkinson's Disease Rating Scale.

When the analysis was restricted to complete cases (*n* = 1,170, 258 ICD events, 22.1%), the inverse association between PNI and ICDs remained significant in the fully adjusted model (OR = 0.95, 95% CI: 0.92–0.99, *P* = 0.005). Similarly, after excluding participants with abnormal liver function (*n* = 1,217, 267 ICD events, 21.9%), the association persisted (OR = 0.94, 95% CI: 0.91–0.98, *P* = 0.002). Exclusion of participants with abnormal creatinine values (*n* = 1,277, 291 ICD events, 22.8%) also yielded consistent findings (OR = 0.95, 95% CI: 0.92–0.98, *P* = 0.002).

Furthermore, we applied several propensity score-based methods to minimize potential confounding bias. The standardized mean differences (SMDs) before and after propensity score matching are presented in Supplementary Figure 1, which demonstrated that covariate imbalance was substantially reduced after matching. In the fully adjusted model, the inverse probability of treatment weighting (IPTW) gave an OR of 0.75 (95% CI: 0.57–0.97, *P* = 0.028). The standardized mortality ratio weighting (SMRW) produced an OR of 0.74 (95% CI: 0.57–0.97, *P* = 0.027). Pairwise algorithmic weighting (PA) method yielded ORs of 0.73 (95% CI: 0.55–0.97, *P* = 0.032). Collectively, all sensitivity analyses confirmed the robustness of the primary finding that higher PNI is associated with lower odds of ICDs in patients with early-stage Parkinson's disease.

### Subgroup analyses

**Figure 3:**
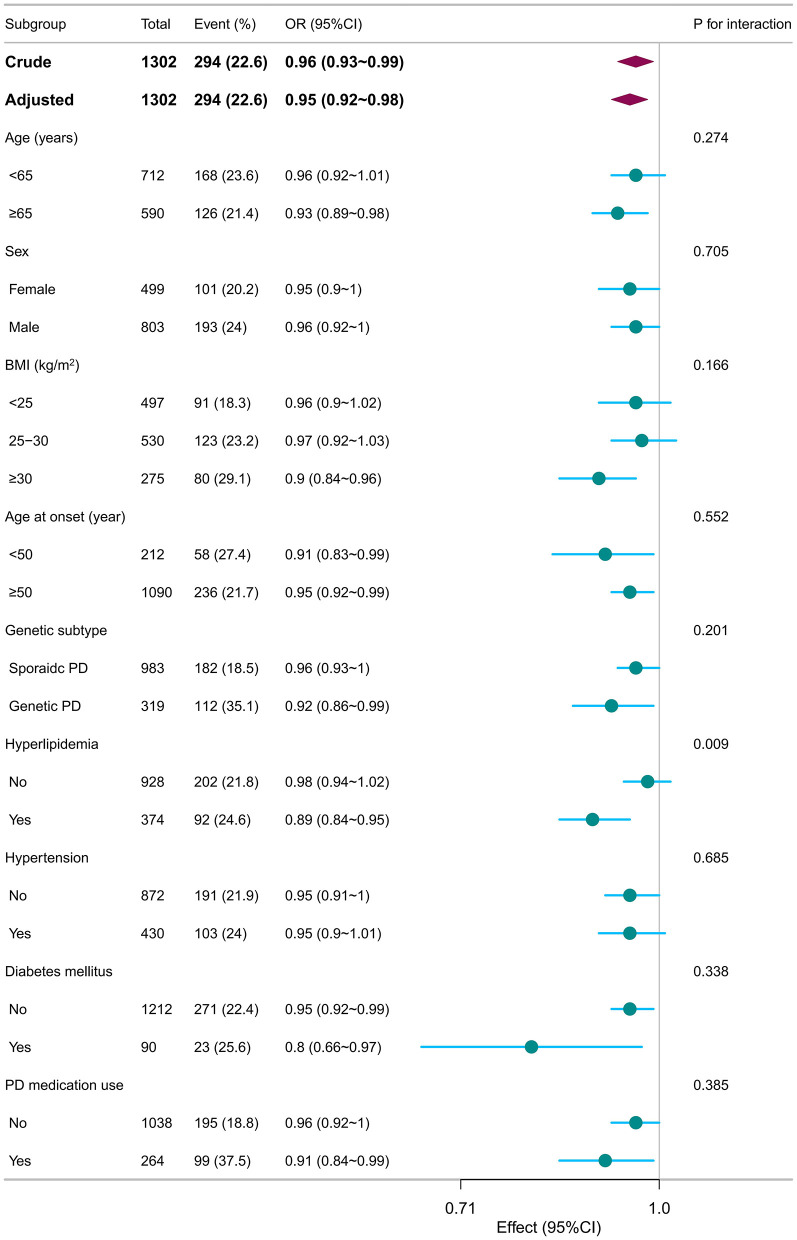
Forest plot of subgroup analyses for the association between PNI and ICDs. Odds ratios (ORs) and 95% confidence intervals (CIs) represent the change in the odds of ICDs per 1-unit increase in the Prognostic Nutritional Index (PNI) as a continuous variable. Except for the Crude model (no covariate adjustment), all other models were adjusted for all covariates in Model 4 (the full adjustment set) except for the stratification variable itself. Model 4 includes: age, sex, education years, race, body mass index (BMI), PD medication use, age at onset, UPDRS Part III score, striatal binding ratio, MoCA score, RBD score, GDS score, hyperlipidemia, hypertension, diabetes mellitus, serum uric acid, AST, and creatinine. The size of each square is proportional to the subgroup sample size. Horizontal lines represent 95% confidence intervals. Interaction *P*-values were derived from likelihood ratio tests comparing models with and without the interaction term. PD, Parkinson's disease; BMI, body mass index; OR, odds ratio; CI, confidence interval. P for interaction values are uncorrected; after FDR correction across all tested interactions, no *P* remained <0.05.

As shown in Figure 3, the inverse association between PNI and ICDs was generally consistent across most prespecified subgroups, including age, sex, age at onset, genetic subtype, PD medication use, hyperlipidemia, hypertension, and diabetes mellitus (all *P* for interaction > 0.05), with the exception of hyperlipidemia, which showed a significant interaction (*P* for interaction = 0.009). Although interaction *P* value of hyperlipidemia was <0.05 before correction, none remained significant after FDR adjustment for multiple comparisons.

### Incremental predictive value of PNI

We further assessed whether adding PNI to the conventional model improved its discriminative performance using ROC curve analysis, with PNI examined both as a continuous variable and as tertiles. The corresponding ROC curves are presented in Supplementary Figure 2 (continuous PNI) and Supplementary Figure 3 (PNI tertiles). The results are summarized in Table 4. Adding PNI as a continuous variable marginally increased the C-statistic from 0.714 to 0.720 (*P* = 0.223 by DeLong's test), and adding PNI as tertiles gave a similar increase from 0.714 to 0.720 (*P* = 0.186). Neither improvement reached statistical significance. Despite the modest and non-significant increases in C-statistics, both reclassification indices showed significant improvements. For the continuous PNI model, the continuous NRI was 0.14 (95% CI: 0.0105–0.2695, *P* = 0.03404) and the IDI was 0.0069 (95% CI: 0.0021–0.0116, *P* = 0.00497). For the tertile-based PNI model, the continuous NRI was 0.1633 (95% CI: 0.0452–0.2813, *P* = 0.00671) and the IDI was 0.0056 (95% CI: 0.0012–0.0099, *P* = 0.01244). These findings indicate that while adding PNI did not significantly increase the AUC, it did provide statistically significant improvement in category-free reclassification and discrimination beyond conventional factors.

**Table 4:** Incremental predictive value of PNI added to the conventional model: C-statistics, NRI, and IDI.

Model	C-statistic (95% CI)	*p*	NRI (95% CI)	*p*	IDI (95% CI)	*p*
Baseline model	0.714 (0.681, 0.747)	Ref.	Ref.		Ref.	
+ PNI (continuous)	0.720 (0.687, 0.753)	0.223	0.14 (0.0105–0.2695)	0.03404	0.0069 (0.0021–0.0116)	0.00497
+ PNI (tertiles)	0.720 (0.687, 0.753)	0.186	0.1633 (0.0452–0.2813)	0.00671	0.0056 (0.0012–0.0099)	0.01244

The baseline model included all covariates from the fully adjusted model (Model 4) except PNI. The extended models added PNI either as a continuous variable or as tertiles (T1 <50.1, T2 50.1–53.85, and T3 ≥ 53.85). CI, confidence interval; IDI, integrated discrimination improvement; NRI, net reclassification improvement; PNI, prognostic nutritional index.

## Discussion

In this large-scale, cross-sectional study of patients with early-stage PD, we first showed that higher PNI was independently associated with lower odds of prevalent ICDs, with each one-unit increment in PNI corresponding to a 5% reduction in odds (OR = 0.95, 95% CI: 0.92–0.98, *P* = 0.003). Further exploratory subgroup analyses revealed no significant interactions across most strata. Adding PNI to baseline models significantly improved category-free reclassification and discrimination, as reflected by the continuous NRI (0.14, *P* = 0.03404) and IDI (0.0069, *P* = 0.00497), despite a modest and non-significant increase in the C-statistic. These observations may have important implications for understanding the association between nutritional-immune status and ICDs in PD.

Previous studies have demonstrated that, compared with healthy controls, patients with obsessive-compulsive disorder (OCD) exhibit significantly elevated neutrophil-to-lymphocyte ratio (NLR) and platelet-to-lymphocyte ratio (PLR) (27–29). Logistic regression analyses further confirmed that both NLR and PLR were significant predictors of OCD (29). In addition, the proportion of regulatory T (Treg) cells was markedly reduced in OCD patients compared with healthy controls (1.0 ± 0.7 vs. 1.9 ± 1.4; *P* = 0.03, r = 0.33) (30), providing additional support for immune imbalance in impulse control spectrum disorders. Beyond these neuropsychiatric conditions, emerging evidence has linked PNI itself to clinical outcomes in PD. One study (31) reported that lower PNI was significantly associated with a higher prevalence of PD (OR = 0.702, 95% CI: 0.515–0.957, *P* = 0.026), and that higher PNI levels were associated with reduced mortality risk (OR = 0.723, 95% CI: 0.674–0.775, *P* < 0.001). Moreover, accumulating research supports the use of PNI assessment for prognostic prediction in individuals with poorer cognitive performance (32). PNI has shown promise as a reliable predictor of the odds of cognitive dysfunction in the elderly population. However, the association between PNI and specific neuropsychiatric endpoints, particularly ICDs, had not been previously examined in early-stage PD. Our findings extend this literature by demonstrating an independent association between PNI and ICDs in drug-naïve early-stage PD.

Several biological pathways may explain the observed inverse association between PNI and ICDs. First, from a pathophysiological perspective, oxidative stress and neuroinflammation are considered core drivers of dopaminergic neurodegeneration in PD (33, 34). Excessive production of reactive oxygen species (ROS) by mitochondria leads to lipid peroxidation, protein and DNA damage, and ultimately neuronal death, while sustained microglial activation maintains a chronic pro-inflammatory environment that accelerates these processes (35). Neuroinflammation can directly or indirectly exacerbate oxidative stress in the brain, rendering neurons vulnerable to damage and death (35). Second, accumulating evidence supports a pathogenic role of neuroinflammation in psychiatric disorders, including major depression, bipolar disorder, schizophrenia, and OCD (10), and ICDs share similar immuno-inflammatory underpinnings. Indeed, transcriptional alterations related to intermittent explosive disorder (IED) have been shown to involve CD4+ T cells, CD8+ T cells, and B lymphocytes (36). Third, low PNI reflects both hypoalbuminemia and lymphopenia. The level of lymphocytes serves as an indicator of the individual's immune system function; a decrease in lymphocyte levels may indicate immune suppression or an underlying health condition, while an increase may suggest an active immune response to infection or inflammation (37). Albumin possesses antioxidant and anti-inflammatory properties, and its reduction may compromise the brain's ability to counteract oxidative damage (38). Furthermore, alterations in serum albumin levels may influence the accumulation of amyloid-β (Aβ) plaques (38). Recent studies have shown that serum albumin levels are negatively correlated with Aβ deposition and Aβ positivity (39). Given that cognitive impairment is closely linked to both PD progression and ICD manifestation, the role of PNI in modulating Aβ pathology may represent an additional mechanistic pathway connecting low PNI to increased ICD risk. The interpretation of albumin in early-stage PD requires caution. While advanced disease is frequently complicated by protein-energy malnutrition (40), *de novo* patients may retain intact nutritional-metabolic profiles (41, 42). It is conceivable that in some individuals, altered dietary patterns could reflect impulsivity-related behaviors; however, our finding of an inverse PNI-ICD association suggests that systemic nutritional-immune status, as captured by the composite PNI, exerts a net protective effect that supersedes any potential behavioral confounding at the individual dietary component level. Concurrently, reduced lymphocyte counts may indicate impaired adaptive immunity, which could facilitate unchecked neuroinflammatory responses. In line with this notion, lower uric acid-to-creatinine ratio (UA/Cr)—another marker linked to oxidative stress—has been selectively associated with specific ICD subtypes and with the coexistence of multiple ICDs in PD patients (13), further supporting the involvement of oxidative and inflammatory pathways in ICD pathogenesis. Collectively, these mechanisms suggest that a lower PNI may serve as a peripheral signature of heightened central neuroinflammation and oxidative stress, thereby increasing susceptibility to ICDs in PD patients. Nevertheless, the modest effect size (7% lower odds per unit increment) and the lack of significant improvement in C statistic indicate that PNI explains only a fraction of ICD variance. This is consistent with the multifactorial etiology of ICDs, where dopaminergic dysfunction, genetic susceptibility, and environmental factors likely contribute substantially. PNI may thus be most valuable as part of a multimodal discriminative assessment rather than as an isolated discriminatory variable.

As an exploratory extension of our findings, emerging evidence implicates the gut-brain-immune axis in PD pathophysiology. Gut dysbiosis influences neurotransmitter synthesis, HPA axis activity, and neuroinflammation (43), while bacterial metabolites such as D-lactate and ammonia may access the CNS via the vagus nerve (44, 45). Notably, the study directly examining gut microbiota in PD patients with ICDs identified enrichment of Methanobrevibacter and Intestinimonas butyriciproducens in impulsive patients, alongside altered metabolic pathways including nicotinate/nicotinamide metabolism and indole alkaloid biosynthesis (46). These taxonomic and functional shifts may disrupt short-chain fatty acid signaling, tryptophan-serotonin metabolism, and adenosine-dopamine interactions (47–50). We hypothesize that PNI may serve as an indirect biomarker of gut-brain axis integrity: hypoalbuminemia may reflect impaired intestinal barrier function and increased bacterial product translocation, while lymphopenia may indicate chronic low-grade inflammation driven by dysbiosis (51). This aligns with the immune-microbiome-brain framework, wherein microbiota regulate cytokine production and microglial function within cortico-striatal circuits (52) implicated in impulse control. Thus, the inverse PNI-ICD association may additionally represent a downstream signature of gut-brain-immune axis dysfunction. However, as the PPMI database lacks microbiome or permeability data, this mechanism remains hypothetical and requires direct validation. We emphasize that this framework is presented as a hypothesis-generating extension rather than an empirically supported pathway, given the absence of microbiome or metabolomic data in PPMI. Its inclusion aims to stimulate future research directions rather than to bolster the clinical validity of PNI, which our primary analyses suggest is modest.

This is the first study to evaluate the association between PNI and ICDs in early-stage PD using a large cross-sectional cohort with comprehensive covariate adjustment and multiple sensitivity analyses. Unlike previous studies (18, 22) that primarily focused on anti-parkinsonian medications as the main driver of ICDs, we adjusted for medication use as a covariate and performed subgroup analyses, confirming that the PNI-ICD association is independent of dopaminergic therapy. This finding suggests that nutritional-immune status may be an independent correlate of ICDs, providing a simple and easily obtainable biomarker for early risk identification in clinical practice.

Nevertheless, several limitations of this study should be acknowledged. First, the cross-sectional design precludes any causal inference regarding the direction of the association between PNI and ICDs. Second, although we attempted to adjust for all relevant potential confounders in the multivariable models, it is possible that some unmeasured or unknown residual confounders may exist (e.g., smoking, alcohol use, caffeine intake, acute infection or inflammatory status, and use of anti-inflammatory medications, dietary habits, family income, and socioeconomic status), which could have led to an overestimation of the observed associations. Third, all participants were recruited from the PPMI cohort, which predominantly includes individuals of White ethnicity, potentially limiting the generalizability of our findings to other racial or ethnic groups. Fourth, although we have proposed the gut-brain-immune axis as a plausible mechanistic framework, the PPMI database does not include direct measures of gut microbiota composition, intestinal permeability biomarkers, or serum metabolomics; consequently, the microbiome-related pathways discussed remain hypothetical and require direct empirical validation. Fifth, PNI was assessed at a single baseline time point; we were therefore unable to evaluate whether intraindividual fluctuations or longitudinal declines in PNI confer differential associated ICD prevalence. Sixth, ICDs were assessed by the QUIP screening questionnaire rather than structured clinical interview or formal DSM-5 diagnostic criteria. While QUIP has been validated for detecting ICDs in PD populations, it may yield higher prevalence estimates compared to diagnostic interviews and cannot establish definitive diagnoses. Our findings should be interpreted as associations with screened ICD symptoms rather than confirmed clinical diagnoses. Seventh, the subgroup analyses were *post hoc* and exploratory, without adjustment for multiple comparisons. The observed hyperlipidemia interaction is hypothesis-generating only, given the small HLP subgroup (*n* = 374) and risk of overfitting, and replication in larger independent cohorts is required. Future studies should incorporate longitudinal designs with repeated measurements of PNI and ICD outcomes, include more diverse ethnic populations, and employ validated diagnostic assessments to confirm and extend our findings. Furthermore, integrating gut microbiome profiling, targeted metabolomics, and intestinal barrier biomarkers, and comprehensive recording of immune-relevant exposures (e.g., acute infections, anti-inflammatory drug use) with PNI trajectories would help disentangle whether the gut-brain-immune axis mediates the relationship between nutritional-immune status and ICDs in PD. Experimental and interventional studies are also warranted to establish causality and explore whether nutritional or microbiome-targeted strategies could mitigate ICD burden.

## Conclusion

In this cross-sectional study of early-stage PD patients, higher PNI was independently associated with lower odds of prevalent ICDs, and adding PNI to baseline models significantly improved category-free reclassification. Longitudinal studies are needed to establish causality and explore potential interventions.

## Data Availability

Publicly available datasets were analyzed in this study. This data can be found here: The data supporting the findings of this study are available from the Parkinson's Progression Markers Initiative (PPMI) database. Access to the de-identified individual-level data is provided to qualified researchers through the Laboratory of Neuro Imaging (LONI) Image and Data Archive (IDA) at https://ida.loni.usc.edu/pages/access/studyData.jsp?project=PPMI, following registration and approval of a Data Use Agreement. The PPMI data repository can also be accessed via https://www.ppmi-info.org/access-data-specimens/download-data.

## Supplementary material

Supplementary Figure 1 Standardized mean difference (SMD) of variables before and after propensity score matching and weighting.

Supplementary Figure 2 ROC curves comparing baseline model (blue) and extended model with continuous PNI (red). Baseline model (blue line) includes all covariates from Model 4 except PNI: age, sex, education years, race, body mass index (BMI), genetic subtype, age at onset, UPDRS Part III score, striatal binding ratio, MoCA score, RBD score, GDS score, hyperlipidemia, hypertension, diabetes mellitus, serum uric acid, AST, and creatinine. Extended model (red line) adds the Prognostic Nutritional Index (PNI) as a continuous variable. Area under the ROC curve (AUC) with 95% confidence intervals: baseline model = 0.714 (0.681, 0.747); extended model = 0.720 (0.687, 0.753). The DeLong test for the difference between AUCs gave *P* = 0.223. The light gray diagonal line represents the reference line (AUC = 0.5).

Supplementary Figure 3 ROC curves comparing baseline model (blue) and extended model with tertiles PNI (red). Baseline model (blue line) includes all covariates from Model 4 except PNI: age, sex, education years, race, body mass index (BMI), genetic subtype, age at onset, UPDRS Part III score, striatal binding ratio, MoCA score, RBD score, GDS score, hyperlipidemia, hypertension, diabetes mellitus, serum uric acid, AST, and creatinine. Extended model (red line) adds the Prognostic Nutritional Index (PNI) categorized into tertiles. Area under the ROC curve (AUC) with 95% confidence intervals: baseline model = 0.714 (0.681, 0.747); extended model = 0.720 (0.687, 0.753). The DeLong test for the difference between AUCs yielded *P* = 0.186. The light gray diagonal line represents the reference line (AUC = 0.5).
