# Supplementary figures and images for: Association between the prognostic nutritional index and impulse control disorders in patients with early-stage Parkinson's disease
Source: Front Nutr. 2026 Jul 17;13:1863248. doi: 10.3389/fnut.2026.1863248 (PMC13426278; doi:10.3389/fnut.2026.1863248)

variable

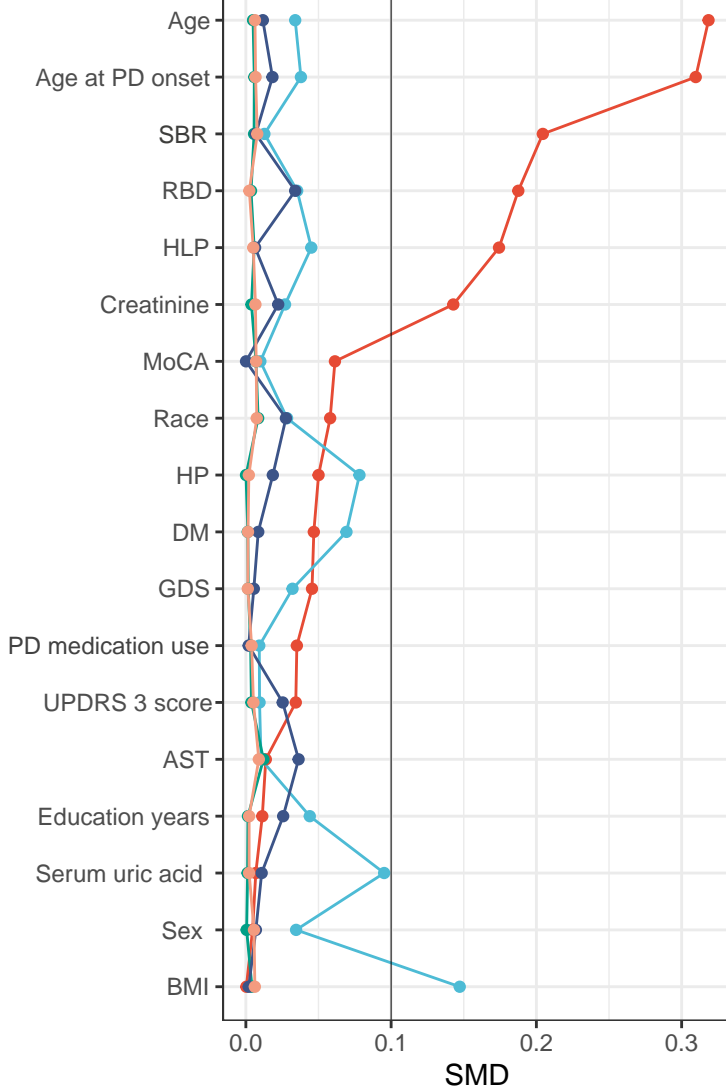

Method

- Unmatched
- Matched
- WeightedIPTW
- WeightedSMRW
- WeightedPA

Supplement: Supplementary Figure 1 — Standardized mean difference (SMD) of variables before and after propensity score matching and weighting. [file Data_Sheet_1.pdf]

## Statistical comparison

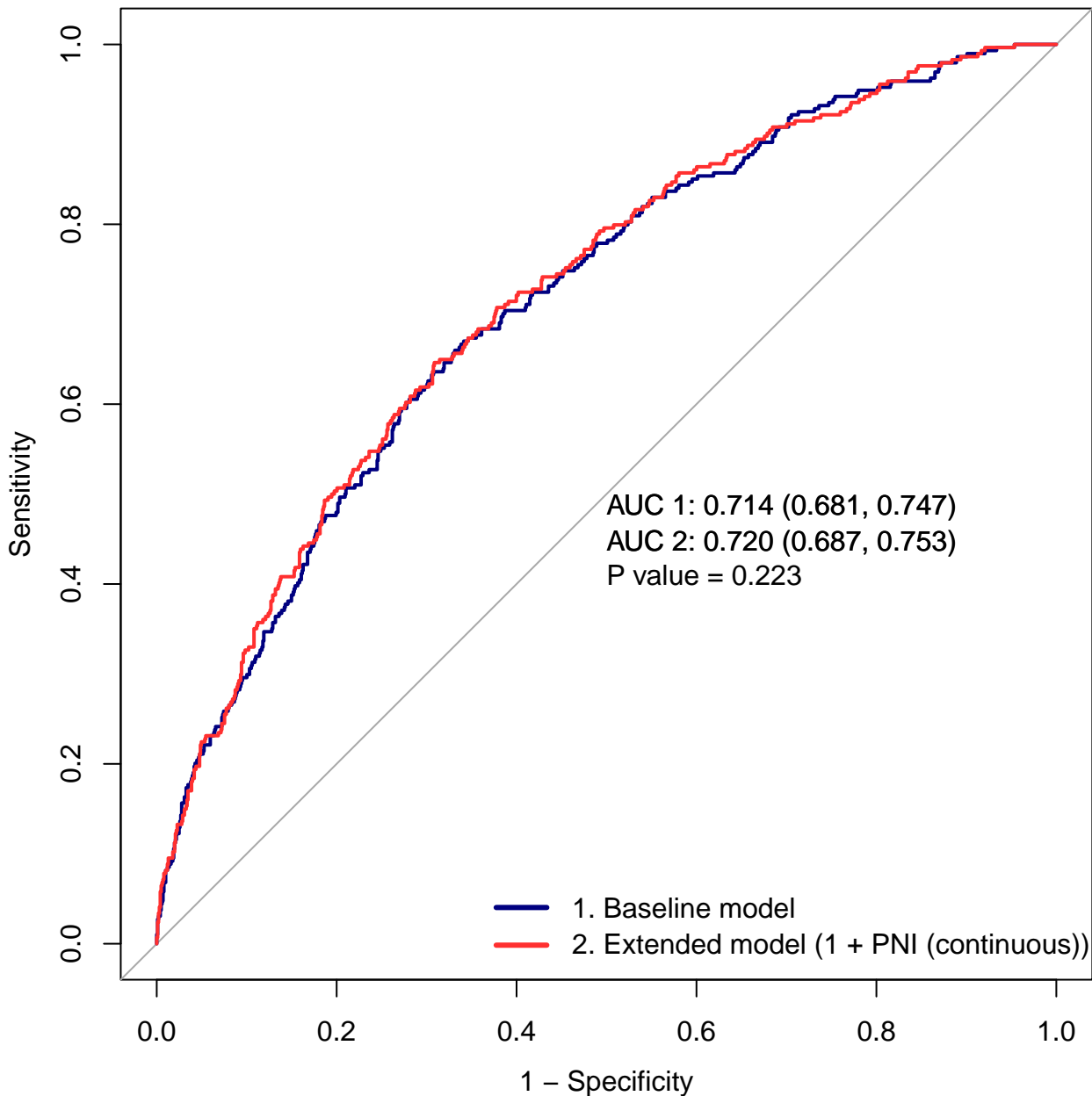

Supplement: Supplementary Figure 2 — ROC curves comparing baseline model (blue) and extended model with continuous PNI (red). Baseline model (blue line) includes all covariates from Model 4 except PNI: age, sex, education years, race, body mass index (BMI), genetic subtype, age at onset, UPDRS Part III score, striatal binding ratio, MoCA score, RBD score, GDS score, hyperlipidemia, hypertension, diabetes mellitus, serum uric acid, AST, and creatinine. Extended model (red line) adds the Prognostic Nutritional Index (PNI) as a continuous variable. Area under the ROC curve (AUC) with 95% confidence intervals: baseline model = 0.714 (0.681, 0.747); extended model = 0.720 (0.687, 0.753). The DeLong test for the difference between AUCs gave P = 0.223. The light gray diagonal line represents the reference line (AUC = 0.5). [file Data_Sheet_2.pdf]

## Statistical comparison

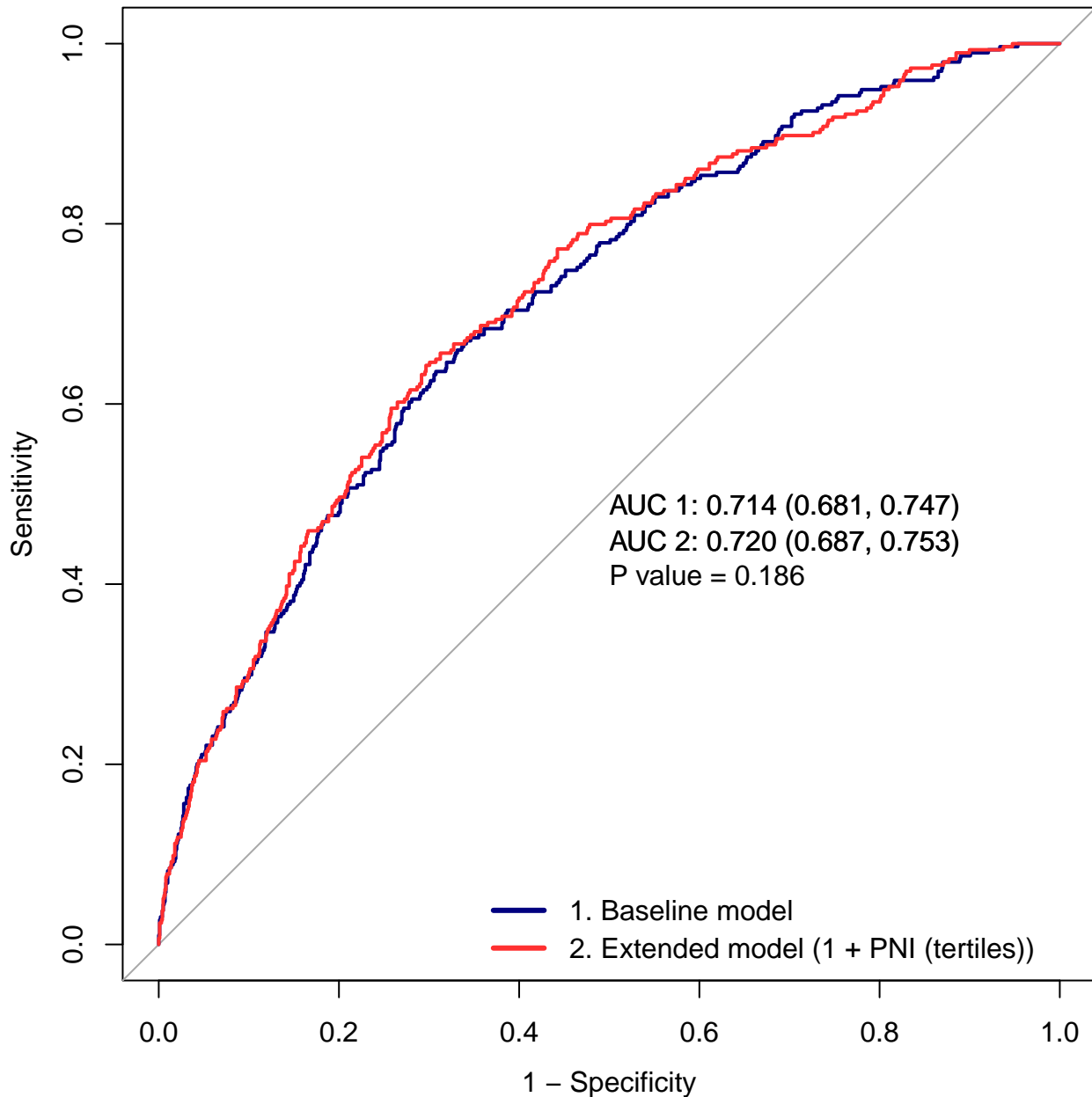

Supplement: Supplementary Figure 3 — ROC curves comparing baseline model (blue) and extended model with tertiles PNI (red). Baseline model (blue line) includes all covariates from Model 4 except PNI: age, sex, education years, race, body mass index (BMI), genetic subtype, age at onset, UPDRS Part III score, striatal binding ratio, MoCA score, RBD score, GDS score, hyperlipidemia, hypertension, diabetes mellitus, serum uric acid, AST, and creatinine. Extended model (red line) adds the Prognostic Nutritional Index (PNI) categorized into tertiles. Area under the ROC curve (AUC) with 95% confidence intervals: baseline model = 0.714 (0.681, 0.747); extended model = 0.720 (0.687, 0.753). The DeLong test for the difference between AUCs yielded P = 0.186. The light gray diagonal line represents the reference line (AUC = 0.5). [file Data_Sheet_3.pdf]
